# Toxicity of NiO nanoparticles to soil nutrient availability and herbage N uptake from poultry manure

**DOI:** 10.1038/s41598-021-91080-y

**Published:** 2021-06-02

**Authors:** Ghulam Abbas Shah, Jahangir Ahmed, Zahid Iqbal, Fayyaz-ul- Hassan, Muhammad Imtiaz Rashid

**Affiliations:** 1 Department of Agronomy, Pir Mehr Ali Shah Arid Agriculture University, Rawalpindi, 46300 Punjab Pakistan; 2 Center of Excellence in Environmental Studies, King Abdulaziz University, P.O Box 80216, Jeddah, 21589 Saudi Arabia; 3 Institute of Soil Science, Pir Mehr Ali Shah Arid Agriculture University, Rawalpindi, 46300 Punjab Pakistan

**Keywords:** Carbon cycle, Environmental impact, Element cycles, Pollution remediation

## Abstract

Recently, there is an increasing trend of using metallic nanoparticles (NPs) in agriculture due to their potential role in remediating soil pollution and improving nutrient utilization from fertilizers. However, evidence suggested that these NPs were toxic to the soil life and their associated functions, and this toxicity depended on their dose, type, and size. Here, a dose-dependent (5, 50, and 100 mg kg^−1^ soil) toxicity of NiO NPs on poultry manure (PM: 136 kg N ha^−1^) decomposition, nutrient mineralization, and herbage N uptake were studied in a standard pot experiment. The NPs doses were mixed with PM and applied in soil-filled pots where then ryegrass was sown. Results revealed that the lowest dose significantly increased microbial biomass (C and N) and respiration from PM, whereas a high dose reduced these parameters. This decrease in such parameters by the highest NPs dose resulted in 13 and 41% lower soil mineral N and plant available K from PM, respectively. Moreover, such effects resulted in 32 and 35% lower herbage shoot and root N uptakes from PM in this treatment. Both intermediate and high doses decreased herbage shoot Ni uptake from PM by 33 and 34%, respectively. However, all NPs doses did not influence soil Ni content from PM. Hence, our results indicated that high NPs dose (100 mg kg^−1^) was toxic to decomposition, nutrient mineralization, and herbage N uptake from PM. Therefore, such NiONPs toxicity should be considered before recommending their use in agriculture for soil remediation or optimizing nutrient use efficiency of fertilizers.

## Introduction

The rigorous use of nanotechnology in developing various branches of the economy resulted in enhanced production and utilization of metallic nanoparticles (NPs). Recently this technology is being utilized in building industry, medicine, cosmetics, electronics, protection of the environment and agriculture^1–6^. Therefore, this anthropogenic release of NPs to the environment, causing a severe threat to the various forms of life in different ecosystems. The toxicological effects on soil life, their associated functions, as well as on metabolism and development of the plants are dependent on chemistry, applied concentrations/doses, and size of the NPs^6,7^. The NPs intensive use in fertilizers, soil, or plant systems raised questions over their potential consequences on soil food web and their associated functions^8–11^. For instance, the application of NPs could result in positive and negative effects on soil life and their functions^11,12^. Several studies reported that application of NPs to soil would not affect pH, electrical conductivity or organic matter, irrespective of the NPs application rate/dose^11,13,14^. However, their influence on other soil biota and associated functions varies depending on the dose and type of the metallic NPs^15–17^.

High NPs application dose (1–2 g kg^−1^ soil) can decrease the activity of soil organisms and disturb their associated function of leaf litter decomposition and N mineralization^11^. Likewise, a dose dependent toxicological influence of ZnO NPs were observed by Hu et al.^16^ on cellulose, mitochondria and DNA of earthworms. They concluded that ZnO NPs dose of < 1 g kg^−1^ soil did not induce toxicity or any harm to the aforementioned parameters whereas > 1 g kg^−1^ prove toxic to these earthworm parameters. Ko and Kong^1^ observed dose-dependent effect of NiONPs on bioluminescence activity and found that 10 and 50 mg L^−1^ doses did not influence this activity whereas 50% inhibitory concentration (IC_50_) value for this parameter was observed at NPs dose of 198 mg L^−1^. Similarly IC_50_ of these NPs for the seed germination of *Raphanus sativus* L was 114.2 mg L^−1^^1^. Likewise, 0.2 µg mL^−1^ of NiONPs did not induce oxidative stress to *Artemia salina*, however the stress induced by other NPs doses (1, 10 and 50 µg mL^−1^) was significantly higher than control, also the stress level increased with increasing concentrations of NPs suspension^18^. Similarly, a dose of 20 µg mL^−1^ weekly applied for a period of 17 weeks to tomato seedling did not influence root elongation and plant height, increased stem weight (26.1 vs. 20.5 g) but significantly decreased weight of leaves (12 vs. 25 g) compared to control^19^. Jośko et al.^9^ found that regardless of types (ZnO and NiO), NPs caused mortality to *H. incongruens*. According to them, the level of mortality initiated by ZnO and NiO NPs was 43 and 40%, respectively in sandy soil sediments. Like Ni, ZnO NPs reduced root length of rapeseed, ryegrass and radish when increasing rates of NPs were applied in the soil, here IC_50_ dose was 50 mg L^−1^ for radish and 20 mg L^−1^ for rapeseed and ryegrass^20^. Besides, 10 mg L^−1^ or lower rates of these NPs did not influence root length^20^. In case of NiO NPs, lower doses ≤ 50 mg L^−1^ were not toxic for bioluminescence activity in the soil^1^. Nevertheless, Dimkpa and Bindraban^12^ believed that use of higher NPs application rates in the soil as well as short-term experiments are mainly responsible for the conclusion of NPs eco-toxicity to soil–plant systems therefore, using lower NPs dosage may be safe for the development of efficient fertilizers.

Granting the limited number of studies on dose-dependent response of Ni or NiO nanoparticles which mostly reported the toxicity of these NPs to living organisms and their associated functions^1,9,18,19,21^. A few very recent dose dependent studies on NiO NPs resulted in contrasting effects on soil functions and plant growth and yield^6,15,21–23^. Chahardoli et al.^6^ observed an increase in *Nigella arvensis L.* biomass at the NiONPs dose of ≤ 50 mg L^−1^ but the doses ≥ 100 mg L^−1^ resulted in significant decrease in plant biomass. Manna and Bandyopadhyay^23^ treated root tips of four different species of Allium with seven different concentrations (10–500 mg L^−1^) of NiONPs and observed that even the lowest dose (10 mg L^−1^) of NPs inserted genotoxicity to the Allium species. On the other hand, Adeel et al.^21^ defined that 5, 50 and 200 mg kg^−1^ soil are the low concentrations of NiONPs when they observed NPs influence on growth and reproduction parameters of earthworm species *Eisenia fetida*. They found that the aforesaid low doses did not influence survival, growth rate and reproduction of these adult earthworms. Alternatively, Wang et al.^24^ found that > 5 mg L^−1^ of NiO NPs significantly decreased microbial activity, N and P removal from activated sludge in sequencing batch reactor indicating that greater than aforesaid doses of NPs are very much toxic to microbial activity and their functions. However, to the best of our knowledge no single study is available in the literature, which investigated the dose dependent toxicity of NiONPs to nutrient availability from organic fertilizer in the soil and nutrient uptakes from these fertilizer by crops.

Poultry manure (PM) is an important source of organic matter (~ 85%) and nutrients especially N (3–4%) that improved soil fertility and plant growth after its soil application therefore extensively recycled as soil conditioner^25,26^. Due to high nutrient content, this manure is prone to nutrient losses in the form of gaseous emission and leaching to the environment^27^. Even after anaerobic digestion, this manure became prone to N_2_O emission^28^. Hence, wise management of this nutrient and organic rich resource is major concern for its recycling as soil conditioner in agriculture. Metallic NPs having high reactivity, surface area, and extremely small size may be used as alternatives to mitigate losses of nutrients from chicken manure and may improve its nutrient uptake by crops after its application to soil. Metallic NPs can be utilize for the mobilization of key nutrients efficiently to the crop plant^8,29,30^. Though, use of NPs for improving nutrient utilization efficiency of organic fertilizer or nutrient delivery to the crops when needed is not much studied topic and need to be thoroughly investigated^8,30^. Consequently, metallic NPs can be assessed as a potential candidate for safe utilization of poultry manure in agriculture.

The main goal of current study is to examine the influence of different doses of NiONPs after mixing with PM on nutrients availability as well as herbage N and Ni uptake. We hypothesized that low dose (5 mg kg^−1^) of NiONPs mixed PM will increase the availability of mineral nutrients (N, P, and K) in the soil for plant uptake. This increment in soil nutrients will result in increasing herbage dry matter (DM) yield, N and Ni uptake. Higher doses (50 and 100 mg kg^−1^) of NiONPs will hinder soil nutrient availability from PM, and ultimately will result in decrement of herbage DM and N uptake. To accomplish this, different doses of NiONPs (5, 50 and 100 mg kg^−1^) were mixed with PM and applied to standard pots containing sandy loam soil where ryegrass was sown and crop yield attributes were monitored for a full growing season. Grass was harvested three time during the experiment and crop dry matter yield, N content, Ni along with soil physio-chemical and microbial properties were monitored.

## Results

### CO_2_ emission

**Figure 1:**
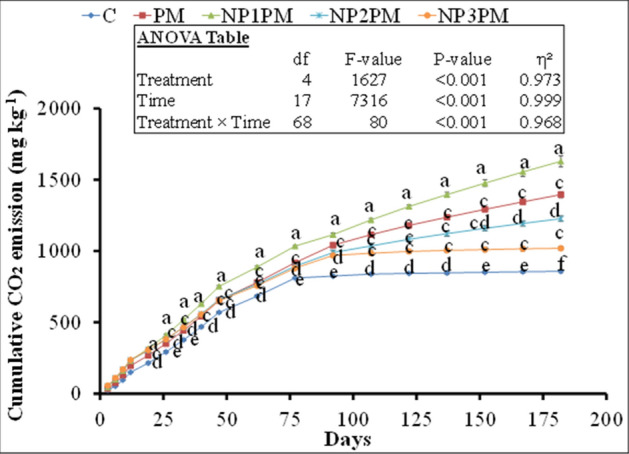
Cumulative CO_2_ fluxes from control (C), poultry manure (PM) and NiO nanoparticles (NP) three doses (NP1 = 5, NP2 = 50 and NP3 = 100 mg kg^−1^) mixed with PM amended soil. NiONPs treatments are abbreviated as NP1PM, NP2PM and NP3PM. Error bars showed standard errors (± 1 SE) of the mean (n = 3). Different small letters illustrated significant differences among treatments at a 5% probability level after the Tukey-HSD test. Inset table represented the outcomes of analysis of variance (ANOVA).

Cumulative CO_2_ emission was significantly affected by the treatments, time and their interaction (P ˂0.001; Fig. 1). After 19 days of the treatments incubation, we did not observe any difference in CO_2_ emission among poultry manure (PM), control and all doses of nickel oxide nanoparticle (NiO NPs) mixed with PM (Fig. 1). However, after 26 days of the manure incubation, all treatments significantly increased CO_2_ emission than control, but NP1PM has higher CO_2_ emission than PM only and there was no difference between PM, NP2PM and NP3PM treatments (Fig. 1). This trend remained the same until day 77 of the incubation. Between 77 and 92 days, the higher doses, NP2 and NP3 treatments significantly decreased CO_2_ emission from PM while NP1 increased this parameter. This decrease was ranged between 5 (989 vs. 1041 mg kg^−1^) to 12% (1227 vs. 1396 mg kg^−1^) for NP2 and 7 (969 vs. 1041 mg kg^−1^) to 27% (1019 vs. 1396 mg kg^−1^) for NP3 treatment during the aforesaid incubation period (Fig. 1). On the other hand, the lowest dose, NP1PM treatment, significantly increased this parameter that was ranged between 7 (1115 vs. 1041 mg kg^−1^) to 17% (1629 vs. 1396 mg kg^−1^) during this incubation period (Fig. 1).

### Influence of nickel oxide nanoparticles amended PM on soil chemical properties

**Figure 2:**
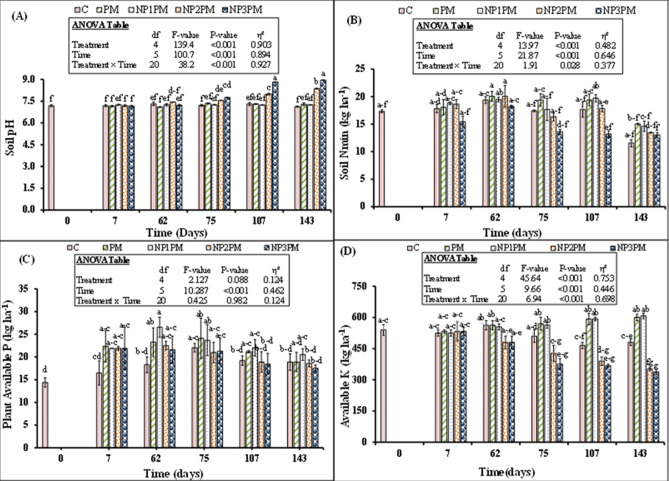
Mean (n = 3) soil pH (**A**), soil mineral nitrogen (Nmin) (**B**), plant available P (**C**), and plant available K (**D**) at 0, 7, 62, 75, 107 and 143 days of sowing ryegrass as affected by sole and combined application of poultry manure (PM) with nickel nanoparticles (NP). Treatments abbreviation can be found in the legend of Fig. 1. Error bars showed standard errors (± 1 SE) of the mean. Different small letters illustrated significant differences among treatments at a 5% probability level after the Tukey-HSD test. Inset table represented the outcomes of analysis of variance (ANOVA).

Soil pH was significantly affected by treatment, time and their interaction (Fig. 2A). Multiple comparison indicates that soil pH was not differed among treatments from day 7 till 62 however this parameter was significantly higher in NP3PM compared to all other treatments. After this time, NP1 and NP2 increased the aforesaid parameter on day 75, 107 and 143 than PM or control (Fig. 2A). This increment was 3, 9, 15% for NP2 and 6, 21 and 23%, respectively for NP3 treatment. However, NP1 amended PM did not influence soil pH.

**Figure 3:**
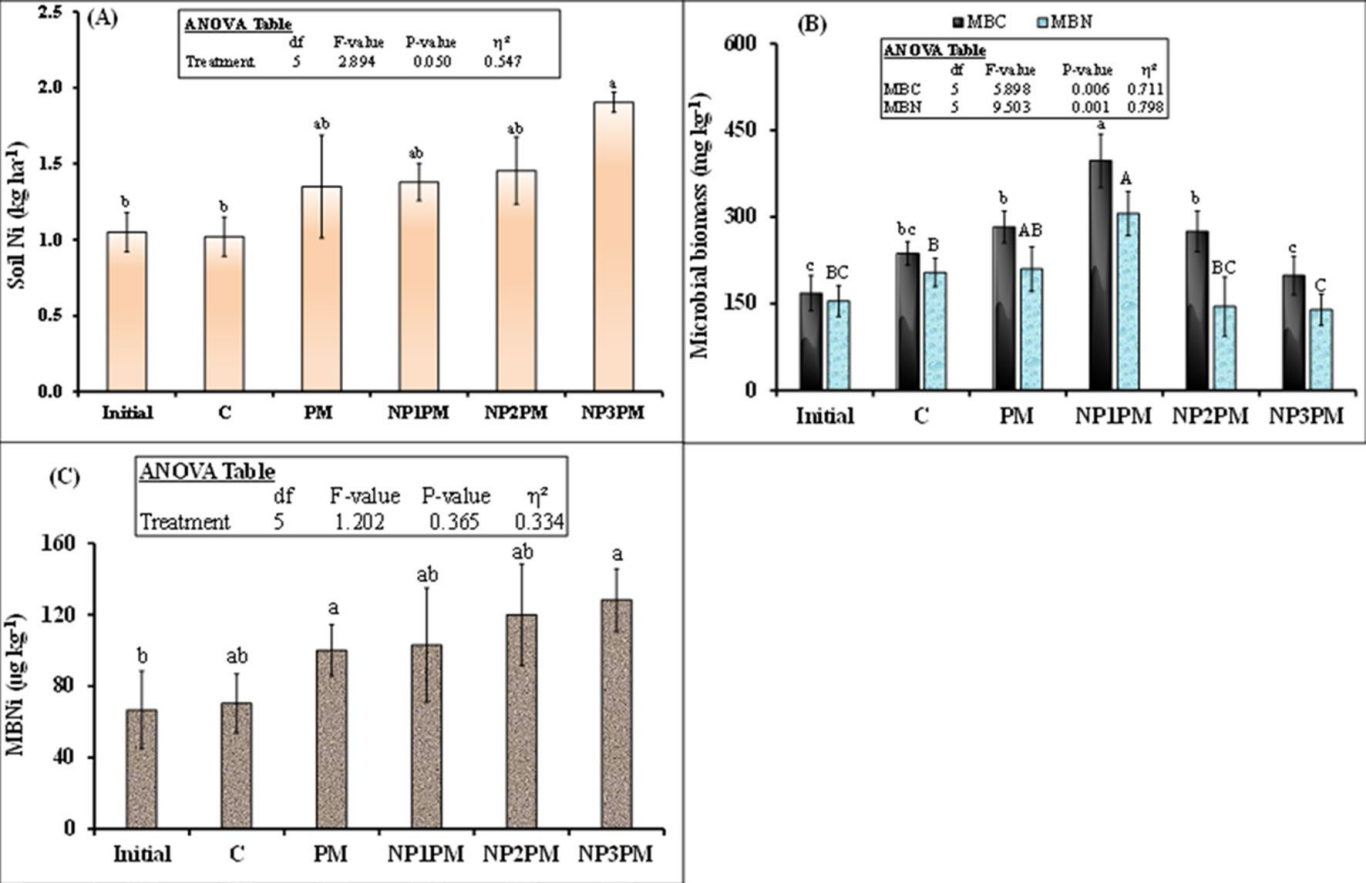
Mean (n = 3) (**A**) soil Ni content after 143 days of sowing ryegrass (**B**) microbial biomass carbon (MBC) and nitrogen (MBN) as well as (**C**) nickel (MBNi) at the start and end of experiment in the soil treated with poultry manure (PM) and three doses of nickel nanoparticles (NP). Treatments abbreviation can be found in the legend of Fig. 1. Error bars showed standard errors (± 1 SE) of the mean. Inset table indicates the analysis of variance (ANOVA). Multiple comparisons among treatments were analyzed by the Tukey-HSD test.

Like soil pH, mineral N was also significantly influenced by treatments, time and their interaction (Fig. 2B). Until day 62, this parameter did not differ among different treatments, but, on day 75, 107 and 143, mineral N from PM was decreased by 30, 32 and 13% by the highest dose of NPs compared to PM only treatment. On the other hand, we did not observe any difference in this parameter between control and NP3PM treatment (P > 0.05) indicating that the highest dose of NiO NPs did not allow microbe to grow and mineralize PM. Lower and intermediate doses (5 and 50 mg kg^−1^ soil) did not affect soil mineral N from PM, therefore we did not observe any difference in soil mineral N among PM, NP1PM and NP2PM treatments. Mineral N in all treatments significantly decreased with time (P˂0.001). Plant available P was neither affected by treatments nor did their interaction with time (P > 0.05), however only time significantly affected this parameter (Fig. 2C; P˂0.001). Generally, plant available P decreased with time in in all PM applied treatments (P˂0.001). Plant available K was significantly influenced by treatments, time and their interaction (Fig. 2D; P˂0.001). Like other nutrients, plant available K content did not differ among treatments on day 7 and 62. On day 75 and later on, both doses (50 and 100 mg kg^−1^ soil) significantly decreased K content in the soil from PM. This decrement was 25, 34 and 31% for NP2 and 34, 38 and 41% for NP3 amended PM than PM alone on day 75, 107 and 143, respectively. The decrease in soil K from PM by both doses of NPs was not different from control. Nevertheless, NP1 did not affect this parameter when mixed with PM (Fig. 2D). All treatments significantly affected Ni content present in the soil at the end of the experiment (Fig. 3A, P = 0.05). Multiple comparison using linear model indicates that all NPs doses did not influence Ni content in the soil compared to PM. However, this parameter was 87% (1.9 vs. 1.0 kg ha^−1^) higher in NP3PM treatment than control and tended to be higher in former treatment compared to PM only (Fig. 3A).

### Microbial biomass carbon, nitrogen and nickel

Microbial biomass carbon (MBC) was significantly affected by the treatments (Fig. 3B; P = 0.006). Interestingly, lowest dose (NP1) of NiO NPs significantly increased MBC compared to PM or control. This increment was 41 (397 vs. 283 mg kg^−1^) and 68% (397 vs. 237 mg kg^−1^) higher than PM and control, respectively. On the other hand, the highest dose (100 mg kg^−1^) of NPs decreased this parameter by 30% (199 vs. 283) than PM but intermediate dose did not influence this parameter compared to PM or control. Similar to MBC, MBN was also significantly affected by the treatments (Fig. 3B; P = 0.001). Again, NP1 increased this parameter by 46 (306 vs. 210 mg kg^−1^) and 50% (306 vs. 204 mg kg^−1^) than PM and control respectively. NP2 did not influence this parameter but NP3 decreased this by 34% (139 vs. 210 mg kg^−1^) compared to PM (P˂0.05). Similar to MBC, MBN was not different among control, PM and NP2PM treatments (Fig. 3B; P > 0.05). Microbial biomass Ni was not significantly affected by the treatments (Fig. 3C). This parameter was tended to be higher in NP2PM than PM only or control. However these effects were not differed significantly among all the studied treatments (Fig. 3C; P > 0.05).

### Plant nitrogen and nickel uptakes

**Figure 4:**
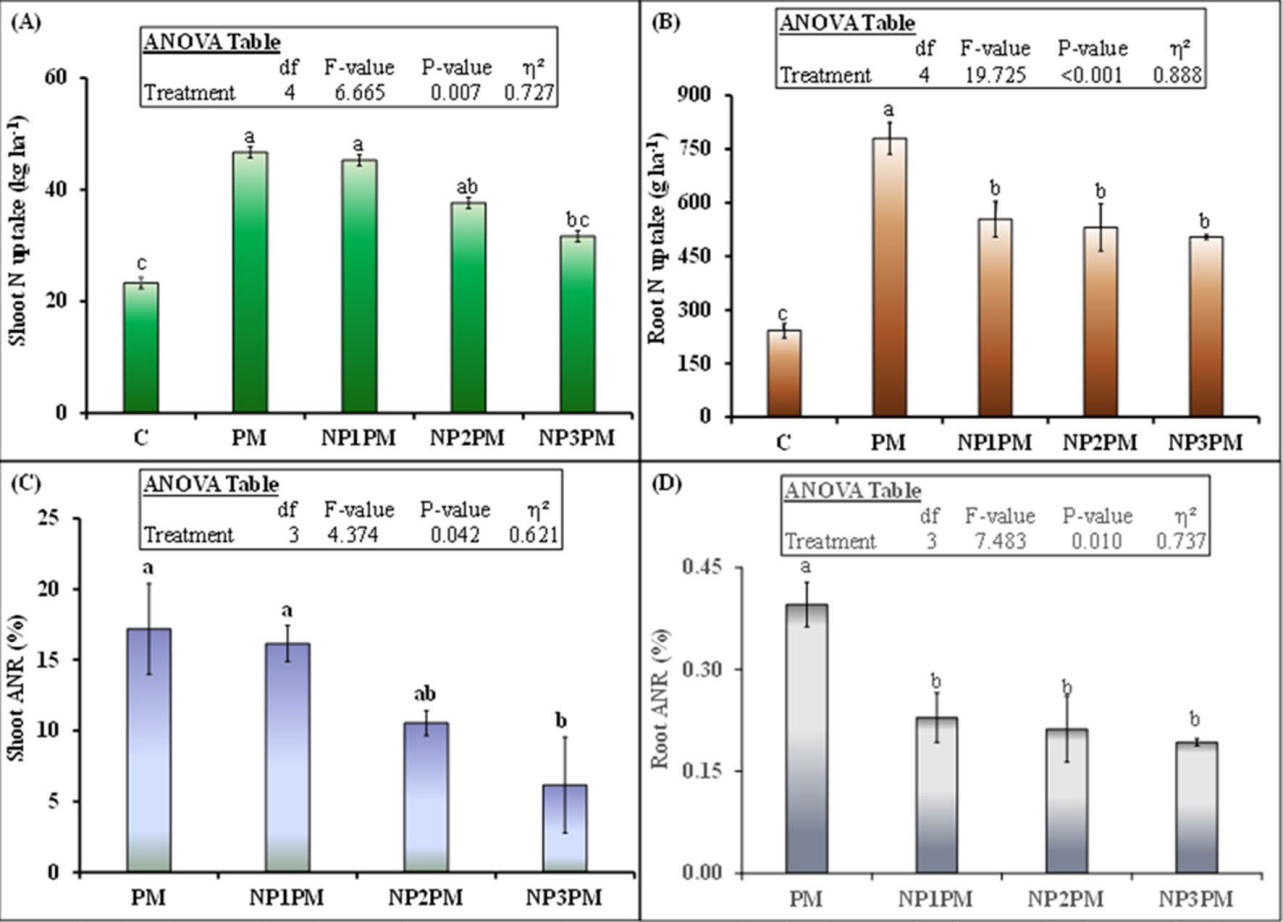
Mean (n = 3) ryegrass (**A**) shoot (cumulative of 3 cuts), (**B**) root N uptake as well as shoot (**C**) and root apparent N recoveries (**D**) after sole and combined application of poultry manure (PM) with nickel nanoparticle (NP). Treatments abbreviation can be found in the legend of Fig. 1. Error bars showed standard errors (± 1 SE) of the mean. Different small letters illustrated significant differences among treatments at a 5% probability level after the Tukey-HSD test. Inset table represented the outcomes of analysis of variance (ANOVA).

All treatments significantly affected root and shoot N uptake of ryegrass (Fig. 4A, B; P = 0.007 and P˂0.001). PM significantly increased root and shoot N uptake than control. This increment was 222 (780 vs. 242 g ha^−1^) and100% (47 vs. 23 kg ha^−1^) higher than control in ryegrass root and shoot, respectively. Among the NPs doses treatments, only the highest NPs dose, decreased shoot N uptake, which was 32% (32 vs. 47 kg ha^−1^) lower than PM alone and did not differ from control. However, shoot N uptake from PM was not significantly different from other doses of NPs (Fig. 4A). In case of root N uptake, all doses of NPs significantly decreased this parameter. This decrement was 29 (554 vs. 780 g ha^−1^), 32 (531 vs. 780 g ha^−1^) and 35% (504 vs. 780 g ha^−1^), respectively than PM only. In contrast to shoot N uptake, this parameter in PM and all NPs doses amended PM was significantly higher than control. This increase was 222, 129, 119 and 108% in PM, NP1PM, NP2PM and NP3PM, respectively (Fig. 4B; P˂0.05). Apparent N recovery (ANR) of both grass organs followed the similar pattern (Fig. 4C, D). There was no difference in ANR among PM, NP1PM and NP2PM. Nevertheless, this parameter was decreased by 64% through highest dose of NPs (Fig. 4C). For root, the highest ANR was observed in PM whereas all doses of NPs decrease this parameter in the root from PM. This decrement was 50 (0.23 vs. 0.40%) in NP1, 100 (0.21 vs. 0.40%) in NP2 and 100% (0.19 vs. 0.40%) in NP3 than PM only (Fig. 4D; P˂0.05).

**Figure 5:**
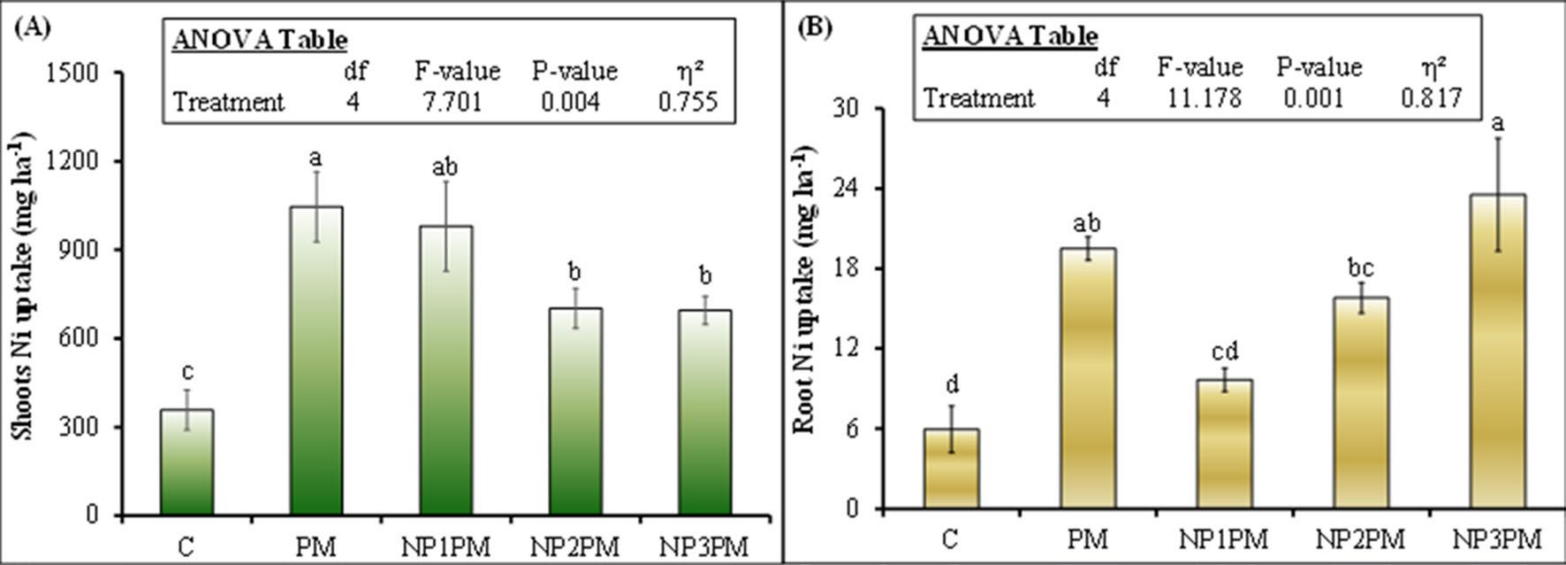
Mean (n = 3) ryegrass (**A**) shoot (total of 3 cuts) and (**B**) root Ni uptake after sole and combined application of poultry manure (PM) with nickel nanoparticle (NP). Treatments abbreviation can be found in the legend of Fig. 1. Error bars showed standard errors (± 1 SE) of the mean. Different small letters illustrated significant differences among treatments at a 5% probability level after the Tukey-HSD test. Inset table represented the outcomes of analysis of variance (ANOVA).

Shoot Ni uptake was also significantly affected by the treatments (Fig. 5A; P = 0.004). PM significantly increased Ni uptake and this increment was 193% (1046 vs. 357 g ha^−1^) in this treatment than control. The lowest NPs dose (NP1) did not affect shoot Ni uptake from the PM, however NP2 and NP3 decreased this parameter by 33 and 34% compared to PM. Besides there was no difference in shoot Ni uptake between NP2 and NP3 treatments (Fig. 5A). Interestingly, in contrast to shoot Ni, root uptake of this parameter was 50% lower in NP1 compared to PM (Fig. 5B). However, the highest dose of NPs (NP3) did not affect root Ni uptake from PM. Moreover, there was no difference in root Ni uptake between NP1 and NP2PM, as well as NP1 and C treatments (Fig. 5B; P > 0.05).

**Figure 6:**
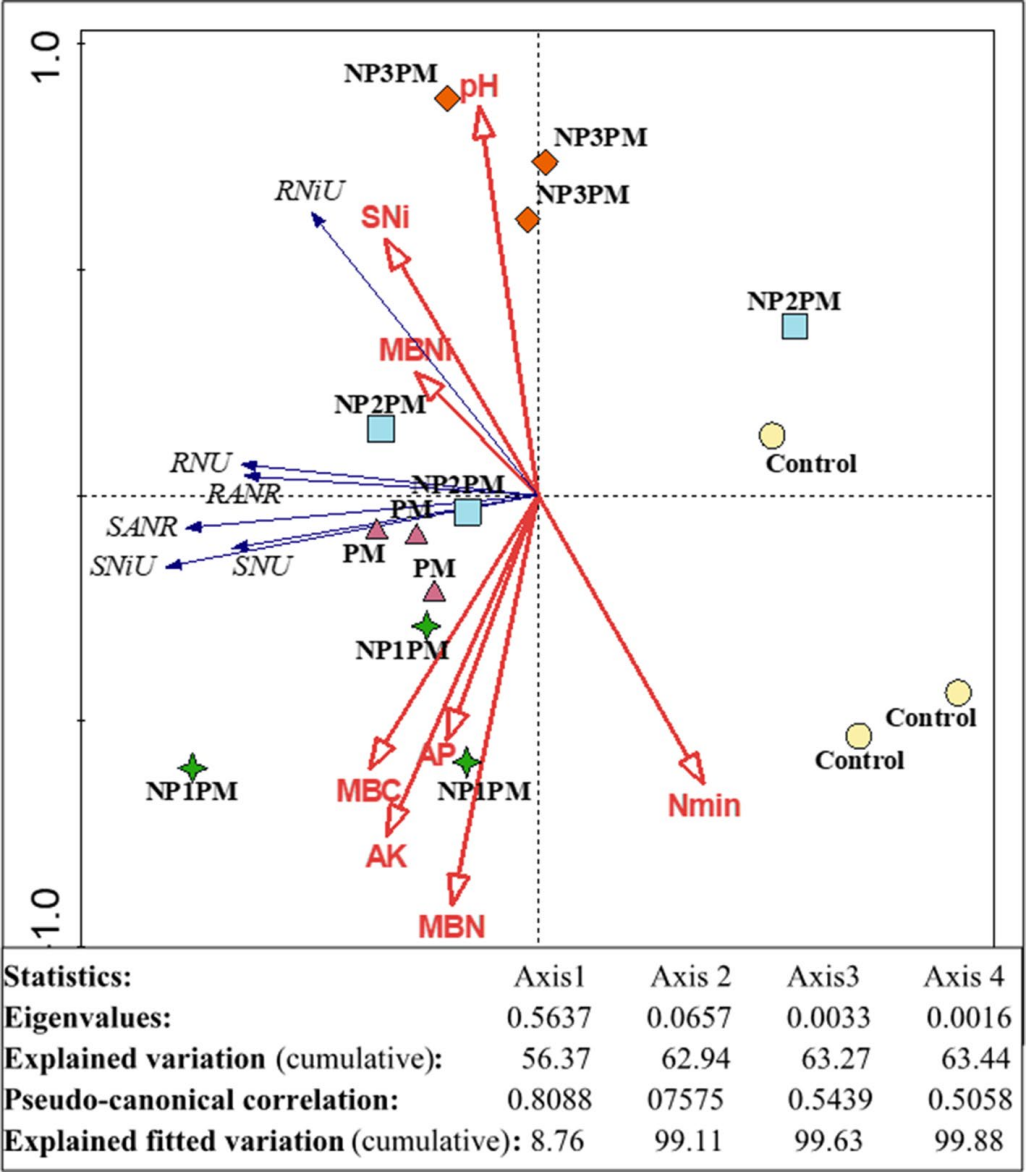
Redundancy analysis (RDA) of soil pH, mineral N (Nmin), nickel (SNi), available P (AP) and K (AK), microbial biomass carbon (MBC), nitrogen (MBN), nickel (MBNi), shoot nitrogen uptake (SNU), Ni uptake (SNiU), apparent N recovery (SANR), root nitrogen uptake (RNU), apparent N recovery (RANR) and Ni uptake (RNU) from control, poultry manure (PM), low dose NiONPs (NP1), intermediate (NP2) and high (NP3) amended PM. Treatments are signified as circle (Control), triangles (PM), stars (NP1PM), squares (NP2PM) and diamond (NP3PM). PC1 (56.3%) and PC2 (62.9%) explained most of the variations in the data with unit less individual scores.

Redundancy analysis (RDA) summarized the relationships of soil pH, mineral N (N_min_), nickel content (SNi), plant available P (AP), K (AK), microbial biomass C (MBC) and N (MBN) with herbage shoot N (SNU) and Ni (SNiU), root N (RNU) and Ni (RNiU) uptakes as well as root (RANR) and shoot apparent N (SANR) recoveries in triplicates of control, poultry manure (PM), NP1PM, NP2PM and NP3PM treatments (Fig. 6). This analysis indicates that most of the soil and plant parameters are inversely related with control and NP3PM treatments. Only, SNi, MBN, pH and SNi are positively associated with NP3PM treatment. The treatments NP1PM and PM are positively associated with SNR, RNR, SNiU, SNU, MBC, MBN, AP and AK treatments. Root and shoot N uptakes are negatively associated with soil Nmin. However, root Ni uptake are positively associated with soil and microbial biomass Ni. Similarly shoot Ni uptake is also positively associated with both of these parameters but this correlation is relatively weaker than RNi uptake (Fig. 6).

## Discussion

Our results are partly in line with our expectation that low dose (5 mg kg^−1^ soil) of NiO NPs enhanced CO_2_ emission and mineral nutrients from PM in the soil and ultimately grass yield and nutrient uptake. We found that CO_2_ emission from the PM was significantly higher after 19 days of incubation and this increment remained higher throughout the laboratory incubation in NP1PM treatment duration till 182 days (Fig. 1). However, the part of the results which did not meet our expectation was that the lower dose (5 mg kg^−1^ soil), which did not increase soil N_min_, plant available P (AP) and K (AK) from PM in the soil throughout the pot experiment (Fig. 2B–D). These results are in accordance with Avila-Arias et al.^15^ who did not find the influence of any low or high doses (11, 211 or 1018 µg Ni g^−1^ soil) of NiO NPs on CO_2_ emission as well as on activities of the β-glucosidase or urease enzymes. They explained that solubility of Ni ions in the soil pore water or their absorption in the dissolved organic matter might be responsible for non-toxic effects of NiONPs on the selected parameters. Rieuwerts et al.^31^ observed that metallic ions are highly soluble in the soil pore water because these ions could make complexes and absorb to the surface of the dissolved organic matter. Correspondingly, PM or its compost can significantly reduce/immobilize Ni in the soil^32,33^. Haroon et al.^32^ found that 10 and 20 t ha^−1^ of PM application reduced Ni concentration by 18 and 33% in the soil, respectively. They explained that organic matter present in PM can immobilize heavy metals in the soil and thus decrease the heavy metal extractability from heavy metal contaminated soil therefore Ni ions availability in the soil may be decreased/halted. In line with these observation, we found no difference in soil Ni content between PM and NP1PM treatments (Fig. 3A). This was also confirmed by very weak correlation between NP1PM, PM and soil Ni content (Fig. 6). Moreover, no increment in N_min_, AP and AK in the soil by NP1 in PM amended soil could also be explained by the higher microbial activity represented by high CO_2_ emission, microbial biomass C and N in our study (Figs. 1, 4A). The higher activity might lead to microbial N, P or K immobilization in the soil during PM decomposition that resulted in reduction of plant available N, P or K in the soil. The microbial biomass acted as a sink of different nutrients in the soil, so microbes can extract substantial amount of nutrients from the soil inorganic nutrient pool to fulfill their energy demand that led to nutrient immobilization^34^. These possible pathways could result in no difference of N_min_, AP and AK content in the soils amended by NP1PM and PM treatments.

Similar to nutrient availability in the soil, low dose of NPs did not influence shoot N uptake and apparent N recovery (ANR) from PM (Fig. 4A, C). However root N uptake or ANR was significantly lower in this treatment than PM alone (4B, D). The no difference in shoot uptake or ANR could be explained by the availability of similar amount of soil mineral N in both NP1PM and PM amended treatments (Fig. 2B). This is despite of the fact that NP1 dose significantly increased microbial biomass C (MBC) but MBN was only tended to be higher and did not differ from PM alone (Fig. 3B; P > 0.05) indicating that there was no difference in microbial N immobilization between two treatments. Decomposition and N mineralization from PM/any organic matter is mainly influenced by microorganisms in the soil^35^. Stark et al.^36^ observed that addition of organic matter (lupin residue) stimulated the microbial activity and resulted in enhanced microbial biomass, enzymatic activities and N mineralization in the soil. Avila-Arias et al.^15^ showed that low or high doses of NiO NPs did not influence microbial activity in unfertilized soil. On the other hand, Aziz et al.^8^ found that lower doses (1.4–2.6 mg kg^−1^ soil) of ZnO NPs increased the mineral N content from biogas slurry. They explained that metallic NPs influenced the soil nutrient availability in many different ways. For example, metallic NPs positively influenced the microbial community and secretion of urease and phosphates enzymes^29,37^ that regulates N and P availability in the soil^38^. However, this effect of NPs on soil microbial communities and their associated functions is metal dependent. Avila-Arias et al.^15^ tested the influence of three metal oxide NPs (Mo, Ni and Li) on soil microbial communities and β-glucosidase and urease enzymes activities. They found that all three NPs used in their study differently affected soil microbial communities and soil enzymes activities. Therefore, NiO NPs in our study may not behave similarly to ZnO NPs in nutrient mobilization and availability in the soil as was the case in our other study by Aziz et al.^8^. Even in our aforesaid study, despite of the increase in N_min_ availability in the soil, ZnO NPs would not able to enhance plant N uptake from biogas slurry. Therefore, this required a detailed mechanistic study to unveil the influence of metallic NPs on the nutrient uptake from organic fertilizers.

Our second hypothesis that high doses (50 and 100 mg kg^−1^) of NiO NPs hinder the decomposition and nutrient availability from PM in the soil, and hence decrease the herbage N uptake from PM. Our results of soil and plant parameters partly supported this hypothesis. Accordingly, we found that CO_2_ emission and AK was significantly decreased by both higher doses of NPs from PM, N_min_ was only decreased by the highest dose (100 mg kg^−1^). According to Wang et al.^24^, the concentration > 5 mg L^−1^ of NiO NPs inhibited the microbial enzymatic activity, decreased the microbial richness and diversity in activated sludge when decomposed in sequencing batch reactor. They explained that after penetration into the microbial cells, the NiO NPs can abolish the equilibrium existed between the oxidation and anti-oxidation processes. They further indicated that high concentrations of NiO NPs (30 and 60 mg L^−1^) facilitated the release of lactase dehydrogenase from activated sludge, which damaged the cytomembrane of microbial cells. Such destructions might be responsible for changes in the physiological functions of microbial communities in the activated sludge, therefore these high concentrations of NiO NPs decreased the removal rate of N and P from the activated sludge^24^. Similar phenomenon could occur in our study, as higher doses of NiO NPs significantly decreased the microbial respiration (Fig. 1), and the highest dose (100 mg kg^−1^) reduced the microbial biomass C and N in NPs mixed PM treatments compared to PM only (Fig. 3B) and therefore this dose of NPs led to decrease N_min_ availability in the soil from PM (Fig. 2B). This was also confirmed by the inverse relationship between NP3PM and N_min_ as shown from redundancy analysis but a weak positive correlation between PM and N_min_ in the soil (Fig. 6). This lower N availability in the soil from PM could result in lower shoot N uptake and apparent N recovery (Fig. 4A, C). In contradictory to our hypothesis, AP from PM was not affected by any dose of NPs (Figs. 1, 2). These results are in accordance with Avila-Arias et al.^15^ who did not observe the influence of high doses, 211 or 1018 µg Ni g^−1^ soil, of NiO NPs on soil enzyme activitities. They explained that solubility of dissolved Ni ions in the soil solution might be the reason of non toxic effects on these parameters. The other reason for such effects could be that the organic matter present in PM can cover the NPs^39^, therefore it avoids the homoaggregation of NPs and hence reduce their bioavailability in the soil^40^. These could be possible reasons of no effects of NiO NPs on soil AP from PM in our study. However, a detailed future mechanistic study is suggested to unveil these effects of NiO NPs on P availability from PM in the soil.

In addition to nutrients availability in the soil for plant growth, crop biomass and nutrient uptake are affected by many other factors when exposed to NiO NPs stress. For instance, Chahardoli et al.^6^ observed that 100 mg L^−1^ dose of NiO NPs decreased many physiological parameters of *Nigella arvensis* plant such as antioxidant activities, DPPH scavenging activity, secondary metabolite formation, as well as total saponin content and antioxidant capacity. These NPs induced oxidative stress through production of reactive oxygen species and malondialdehyde in wheat crop^22^ as well as decrement in the root length and genotoxic effects to *Allium cipa*^23^ resulted in the decreased growth and biomass yield of different crops. Recent studies showed that nano-sized heavy metals and their bulk counterpart shared same mechanisms of phytotoxicity induction in crops^22,41–43^. Accumulation of Ni induced toxicity to growth and various development stages resulted in reduction of wheat yield^22^ as well decreased the roots and leaves biomass of barley crop^43^. Though, the toxicity induced by NiO NPs to the plant physiological attributes is not in the scope of the current study, but we observed decrement in the root growth which was depicted from the lower root biomass and N uptake from all doses of NPs amended PM (Fig. 5B, D), however the shoot biomass and N uptake and ANR was only decreased by the highest dose (100 mg kg^−1^) of NPs whereas the low and intermediate doses did not affect this parameter (Fig. 4A, C).

According to our expectation, shoot Ni uptake was significantly lower in intermediate and high doses of NPs mixed PM compared to PM alone. In contrast to our expectation, the root Ni uptake was significantly lower in NP1PM treatment but it was not different from PM in the intermediate and high doses of NPs amended PM (Fig. 5B). Saleh et al.^22^ observed that NiO NPs increased the Ni concentration in the root and shoot tissues under ambient CO_2_ environment and the accumulated Ni content was much higher in root than shoot. Similar results of Ni accumulation in root of barley crop was observed by Soares et al.^44^. They explained that plant roots developed some mechanism which limits Ni translocation to the leaves, the most important organs of the plant where photosynthesis took place^44^. We also observed the similar pattern in Ni uptake though in contrast to aforementioned studies, this metal content was much lower in grass root than shoot in the current study. However, the multiple comparison among treatments indicated that the intermediate and high doses of NiO NPs mixed PM resulted in significantly lower shoot Ni uptake and root Ni uptake was not different in the aforesaid doses mixed PM treatments compared to PM alone (Fig. 5). This contradiction in our study could be linked to the Ni availability in the soil, since we applied organic matter in the form of PM to the soil which has ability to absorb/immobilize Ni in the soil^32,33^. Additionally, microbes can also immobilize metal in their biomass^45^ and decrease the extractability of Ni from the soil. Consequently, the low availability of Ni could result in lower Ni uptake in the root and shoot in NPs amended PM treatments compared to PM alone. This was also confirmed by very strong positive correlation between soil Ni content as well as roots and shoots Ni uptakes from redundancy analysis (Fig. 6). Moreover, relatively weak correlation existed among microbial biomass Ni, herbage root and shoot Ni uptakes (Fig. 6).

## Conclusions

Our study provides the first evidence of dose-dependent toxicity of NiONPs to poultry manure (i.e. any organic soil amendments) decomposition, nutrient mineralization and its grass N uptake. Our results indicated that the lowest (5 mg kg^−1^) and intermediate doses (50 mg kg^−1^) of NiONPs did not influence most of the studied soil and plant parameters except increased in microbial respiration (CO_2_ emission) and microbial biomass C by the lowest dose and decreased in root N uptake by both the lowest and intermediate doses. Such non-toxic effects of these doses to most of the studied parameter could be ascribed to Ni immobilization by microorganisms as well as fixation on organic matter present in the PM. This indicates that both lowest and intermediate doses were not toxic to most of the studied soil and plant parameters at least for one crop growing season but if application of these doses to the soil will be continued for longer run then these doses may deteriorate soil quality and decrease the crop yield. On the other hand, 100 mg kg^−1^ concentration of NiO NPs significantly decreased most of the studied parameters in the soil from PM. Herbage Ni uptake from PM was also decreased by the intermediate and high doses of NiONPs indicating that these concentrations are toxic for physiological development of the plant. Hence, our results suggested that the 100 mg kg^−1^ NiONPs are toxic to the soil and plant even when organic matter is applied in the soil. Therefore, our study confirmed that continous application of of NiONPs could be toxic to manure decomposition, nutrient mineralization and herbage N uptake. Consequently, concentration related toxicity of these nanoparticles should be considered for their short or long term use when recommending their application in the development of nutrient efficient fertilizers or as a soil remediation technology.

## Materials and methods

### Nickle oxide nanoparticles

Nickle oxide nanoparticles were purchased in the form of powder from Shanghai Pantian Material Co., Ltd. China. These nanoparticles are spherical in shape and has < 100 nm size with a mean particle size of > 30 nm. The purity assay of nanoparticles was 99.98% trace metal basis with bulk density of 0.53 g ml^−1^ (manufacturer information). This material was also utilized by Adeel et al.^21^ who observed their nominal surface area raged between 30 and 50 m^2^ g^−1^ and zeta potential of these nanomaterials was 15. Further detailed characterization of the nanomaterials can be found in Adeel et al.^21^.

### A standard pot study

To study the objective, we performed a standard pot experiment at the research facility of Department of Agronomy, Pir Mehr Ali Shah Arid Agriculture University, Rawalpindi, Pakistan. This is an agricultural farm located at a latitude of 32.9303° N, with longitude 72.8556° E and altitude of *2500 feet* from sea level. Köppen–Geiger classified this region climate as local steppe. Temperature of the region may rise up to 40 °C in summer however, in winter it is cold and range between 4 to 25 °C. Air temperature and rainfall are varied between 8–30 °C and 0–78.4 mm with a mean of 14.5 mm occurred during the experimental duration (university meteriological station).

Clay loam soil that was previously used in Sadaf et al.^46^ collected from the research area of Pir Mehr Ali Shah Arid Agriculture University, Rawalpindi, Pakistan. Fresh soil was sieved to pass through 2 mm mesh screen for removing root debris. Afterward, each pot was filled with 10 kg of the sieved soil. The diameter and depth of each pot was 21 and 24 cm, respectively. Five treatments i.e., (1) Control (C) (no fertilization), (2) Poultry manure alone at recommended dosage of N (PM), (3) PM + 5 mg NiO NPs kg^−1^ of soil (NP1PM), (4) PM + 50 mg NiO NPs kg^−1^ of soil (NP2PM), and (5) PM + 100 mg NiO NPs kg^−1^ of soil (NP3PM) were laid out according to the completely randomized design. The three concentrations of NiONPs were selected based on their positive, no and/or negative effects on organic matter, soil and plant parameters from the literature^6,19,23,24^. Each treatment consisted of three replications. PM was applied in each treatment at application rate of 136 kg N ha^−1^ except control (unfertilized). The manure was collected from the Institute of Livestock and Poultry, Rawalpindi, Pakistan. The chemical composition of PM is presented in Table 1. A seed rate of 0.3 g pot^−1^ was used to sow seed after treatment application in each pot. Then, these pots were arranged at an open field to provide the ryegrass conditions comparable to natural field. They were watered manually using watering cane and increase in moisture content was monitored by the moisture meter (FY-901, Hangzhou FCJ I & E Co., Ltd., China). The moisture content were maintained at 60% water holding capacity^47^.

**Table 1:** Chemical characteristics of soil and poultry manure used in the study.

	DM^b^	OM^c^	TC^d^	Total N	C:N ratio	P	K	Ni	EC^e^	pH
	%					mg kg^−1^			dS m^−1^	
Soil	88.7 ± 0.04	0.68 ± 0.017	0.91 ± 0.06	0.056 ± 0.01	16.3 ± 0.4	3.2 ± 0.2	120 ± 6	0.23 ± 0.03	1.27 ± 0.01	7.2 ± 0.1
PM^a^	72.6 ± 0.9	61.4 ± 1.31	32.5 ± 0.3	2.1 ± 0.1	15.5 ± 0.6	25,467 ± 273	23,267 ± 467	58.3 ± 0.9	5.4 ± 0.1	7.7 ± 0.1

^a^Poultry manure, ^b^dry matter, ^c^organic matter, ^d^total carbon, ^e^electrical conductivity.

### Soil chemical analysis

After treatment application, the soil was sampled five times during the experimental period. The first sampling was carried out just before application of NiO NPs and PM. Afterwards, soil sampling was done at 7, 62, 75, 107 and 143 days after sowing of ryegrass. Soil samples from each pot were taken at depth of 0–15 cm from three random locations and thoroughly mixed to get a composite sample. These composite samples were analyzed for mineral N (N_min_), pH, plant available potassium (AK) and phosphorus (AP). Besides, soil Ni, microbial biomass carbon (MBC), nitrogen (MBN) and Ni (MBNi) of initial (before application of treatments) and final samples (143 DAS) were analyzed. A multi-meter (inoLab Multi 9430 IDS, WTW, GmbH & Co., KG., Germany) after its standardization with 0.01 N KCl at 25 °C was used to measure soil EC and pH from the prepared solutions^48^. Spectrophotometer was used to determine mineral N (NH_4_^+^–N and NO_3_–N) content from the composite soil samples^49^. To determine this, a 20 g soil was weighed in conical flask from composite sample. Thereafter, 40 mL of ammonium bicarbonate–diethylenetriaminepentaacetic acid (AB-DTPA) solution was mixed in the flask and shaken for 15 min on horizontal electric shaker. Later, in each test tube, 3 mL of both NaOH and CuSO_4_ and 2 mL solution of hydrazine sulfate were added. After shaking, these test tubes were heated at 38 °C in a water bath for a period of 20 min. Subsequently, nitrate color developing reagent (3 mL) was mixed and spectrophotometer was set at 540 nm to measure the absorbance. To determine NH_4_^+^–N from the same extract (3.5 mL), sodium phenate (5 mL) and sodium hypochlorite (3 mL) solutions were mixed and then the solution was shaken and heated at 70 °C for 20 min in water bath^50^. Thereafter, spectrophotometer was used to record absorbance of the solution at 600 nm^50^. Plant available P and K in the composite soil samples were measured by the procedure of Houba et al.^51^. Atomic absorption spectrophotometer (Polarized Zeeman, ZA3000 Series, Hitachi, Japan) was used to determine soil Ni content according to the procedure described in Gil et al.^52^.

### Microbial biomass carbon and nitrogen

We used fumigation extraction technique to determine C and N content in the microbial biomass (C_mic_ and N_mic_)^53,54^. A 10 g fresh soil was weighed from each treatment’s composite sample and then divided this soil sub-sample equally into two parts. One part of 5 g soil was fumigated with ethanol free CHCl_3_ at 25 °C for 24 h. The fumigants were removed by placing the soil samples in water bath at 80 °C. After cooling, the mixture of each sample was extracted with 20 mL of K_2_SO_4_ (0.5 M) using a reciprocal shaker for 30 min. Second part of the sample (non-fumigated) was also extracted using aforesaid extraction methodology. From the filtrate, total C (TOC) and total N (Ntot) was determined through TOC analyzer (TNM-1, Shimadzu, Japan) and Kjeldahl digestion procedure^51^, respectively. The C_mic_ or N_mic_ was calculated by using Eq. (1).

1 $${\text{C}}\,{\text{or}}\,{\text{Nmic}} = { }\frac{{{\text{TOC}}\,{\text{or}}\,{\text{N}}tot_{fum} { }{-}{\text{ TOC}}\,{\text{or}}\,{\text{Ntot}}_{non - fum} { }}}{{{\text{kEC}}\,{\text{or}}\,{\text{kEN}}}}$$

where the values 0.45 and 0.54 were used for kEC and kEN coefficients in the calculation of C_mic_^55^ and N_mic_^53,56^, respectively.

### Microbial biomass Ni

Microbial cells were lysed by chloroform (CHCl_3_) to extract Ni present in microbial biomass^45^ using fumigation extraction technique. However, for extraction of MBNi, we used 25 mL NH_4_NO_3_ (1 M) instead of K_2_SO_4_. Suprapur HNO_3_ was used to acidify the filtrate mixture. The filtrate was stored at 4 °C till further analysis. Laterally, atomic absorption spectrometer (Polarized Zeeman, ZA3000 Series, Hitachi, Japan) was used to detect labile Ni content present in the filtrate of both fumigated and non-fumigated samples. The Ni content present in microbial biomass was calculated by subtracting metal content of non-fumigated filtrate from fumigated ones.

### Plant analysis

The ryegrass was harvested from all pots on day 62, 107 and 143 during the whole experiment. The fresh matter yield was determined by weighing the biomass immediately after plant harvest. Subsequently, the samples were placed at 70 °C in an oven for 48 h to quantify dry matter (DM) yield. After grass cutting at final harvest, the whole clump of soil was taken out from the pot and situated in the cold water container for 2 h to separate roots. After soaking, we divided the soil clump into six parts, and a mesh cloth (0.5 mm) was wrapped around the soil clump to separate the roots using tap water jet. After root separation, the roots were air dried and weighed to measure their fresh weight^25^. The samples were dried at 70 °C in an oven for 48 h to determine root DM content.

### Ryegrass N and Ni uptakes from PM

Nitrogen content in plant samples (shoot and root) were determined by Kjeldahl digestion method. For determination of plant Ni content, ground dry material (1 g) was mixed with concentrated H_2_SO_4_ (5 mL) in the glass tubes that were placed in block digester and the plant samples were digested by gradually increasing the digester temperature to 145 °C for 1 h. Thereafter, 5 mL tri-acid mixture was added in the tubes and digester temperature was increased to 240 °C for the next 60 min^51^. Subsequently, the samples were cool down and filtered and Ni content were determined by atomic absorption spectrophotometer (Polarized Zeeman, ZA3000 Series, Hitachi, Japan) and N content through Kjeldahl method.

Plants N and Ni uptakes as well as the apparent N recovery (ANR) were calculated by using following formulas.

2 $${\text{N}}\,{\text{or}}\,{\text{Ni}}\,{\text{uptake}}\,\left( {{\text{kg}}\,{\text{ha}}^{{ - {1}}} { }} \right) = { }(N_{rS} \, or\,Ni_{rS} \times DM_{rS} ) - (N_{0} \,or\,Ni_{0} \times DM_{0} ){ }$$

3 $${\text{ANR}}\,{\text{and}}\,{\text{ANiR}}\,\left( {\text{\% }} \right) = \frac{{\left( {{\text{N}}\,{\text{or}}\,{\text{Ni}}\,uptake} \right)}}{{{\text{TN}}_{a} }}{ } \times 100$$

where N_rs_ or Ni_rs_ is N or Ni content in the root or shoot samples of ryegrass plant (kg N or Ni (Mg DM^−1^) obtained from PM treatment. DM_rs_ designates DM yield (Mg ha^−1^) of the root or shoot samples of ryegrass obtained from PM treatment. N_0_ or Ni_0_ represent N or Ni observed in the plant samples of control treatment. DM_0_ indicates the root or shoot DM yield (Mg ha^−1^) of ryegrass in control and TN_a_ represents the amount of total N (kg ha^−1^) or Ni applied (Mg kg^−1^ of soil) in the form of manure and/or nanoparticles to the pots.

### Incubation experiment for measuring CO_2_ emission

We performed this incubation experiment in 3 L plastic jars, each with surface area of 0.045 m^2^, filled with 1 kg of same aforementioned clay loam soil as used for ryegrass experiment. The root debris from field moist soil removed by sieving it from 2 mm mesh screen before filling the soil into plastic jars. Similar treatments as mentioned above in ryegrass experiment were used, i.e. 1) Control (C) (without fertilization), 2) PM, 3) NP1PM, 4) NP2PM, 5) NP3PM in triplicates. These pots were placed in an open space using Complete Randomized Design (CRD). After PM mixing, NiO NPs were thoroughly mixed in the soil surface by spatula and 60% moisture content were maintained using weight difference method. These jars were placed in the dark chamber kept outside in an open space.

### Measurement of CO_2_

To capture CO_2_, 10 mL NaOH (1 M) was pipetted in a small petri plate and then this plate was positioned in each soil-filled jar_._ After sample collection, freshly prepared NaOH was placed in petri plate and positioned in the jar again at each sampling event. Subsequently, CO_2_ leakage was avoided by sealing the plastic jars with screw lids alongside fixing a self-adhesive tape^11^. In total, NaOH solution was removed for 18 times throughout the incubation period of 182 days. Initially, first four samplings were done each after 3 days interval followed by next five samplings after every week then last 10 samplings were taken after every 15 days. A similar petri dish containing NaOH solution was placed in empty jar (blank) to correct ambient concentration of CO_2_. The excess of NaOH in these samples were back titrated with 1 M HCl solution to measure CO_2_.

### Statistical analysis

Treatments and time effects on plant and soil parameters were statistically analyzed using univariate analysis with statistical software packages SPSS 17.0 (IBM, New York, USA) and Statistix 8.1 (Analytical software, Leon County, Florida). These main effects of treatments and time on soil and plant parameters at different time intervals were analyzed by analysis of variance in univariate linear model. The significance among treatments was tested at 5% probability level. Tukey-HSD test was used to analyze the multiple comparisons among various treatments. The influence of treatments on soil pH, mineral N, nickel, available P and K, microbial biomass carbon, nitrogen, and nickel, shoot N and Ni uptakes, apparent N recovery, root N, Ni uptakes and apparent N recovery from control, poultry manure (PM), and PM amended with three doses of NiONPs; low (NP1PM), intermediate (NP2PM) and high doses (NP3PM) and their correlations were analyzed by redundancy analysis (RDA) using CANOCO 5.0 for Windows software Microcomputer Power Inc. (Ithaca, NY) on correlation matrices.

### Exprimental research guidelines on plants/seeds

The experimental research and field studies on plants/seeds were complied with relevant institutional, national, and international guidelines and legislation.

## Data Availability

The datasets generated during and/or analysed during the current study are available from the corresponding author on reasonable request.
